# Case Report: A Case of Severe Clinical Deterioration in a Patient With Multiple Sclerosis

**DOI:** 10.3389/fneur.2020.00782

**Published:** 2020-08-18

**Authors:** Katharina Breitkopf, Aykut Aytulun, Moritz Förster, Bastian Kraus, Bernd Turowski, Doreen Huppert, Norbert Goebels, Harald Hefter, Orhan Aktas, Imke Metz, Wolfgang Brück, Guido Reifenberger, Hans-Peter Hartung, Philipp Albrecht

**Affiliations:** ^1^German Center for Vertigo and Balance Disorders, Ludwig Maximilian University of Munich, Munich, Germany; ^2^Department of Neurology, Ludwig Maximilian University of Munich, Munich, Germany; ^3^Department of Neurology, Medical Faculty, Heinrich Heine University Düsseldorf, Düsseldorf, Germany; ^4^Department of Neuroradiology, Medical Faculty, Heinrich Heine University Düsseldorf, Düsseldorf, Germany; ^5^Institute of Neuropathology, University Medical Center Göttingen, Göttingen, Germany; ^6^Institute of Neuropathology, Medical Faculty, Heinrich Heine University Düsseldorf, Düsseldorf, Germany

**Keywords:** multiple sclerosis, tumefactive multiple sclerosis, demyelinating disease, multiple sclerosis rebound, immunocompromised multiple sclerosis patient, progressive brain lesions

## Abstract

Tumefactive multiple sclerosis (MS) is a rare variant of MS that may lead to a rapidly progressive clinical deterioration requiring a multidisciplinary diagnostic workup. Our report describes the diagnostic and therapeutic approach of a rare and extremely severe course of MS. A 51-year-old man with an 8-year history of relapsing-remitting MS (RRMS) was admitted with a subacute progressive left lower limb weakness and deterioration of walking ability. After extensive investigations including repeated MRI, microbiological, serological, cerebrospinal fluid (CSF) studies, and finally brain biopsy, the diagnosis of a tumefactive MS lesion was confirmed. Despite repeated intravenous (IV) steroids as well as plasma exchanges and IV foscarnet and ganciclovir owing to low copy numbers of human herpesvirus 6 (HHV-6) DNA in polymerase chain reaction (PCR) analysis, the patient did not recover. The clinical presentation of tumefactive MS is rare and variable. Brain biopsy for histopathological workup should be considered in immunocompromised patients with rapidly progressive clinical deterioration with brain lesions of uncertain cause.

## Introduction

Multiple sclerosis (MS) is a chronic inflammatory demyelinating disease of the central nervous system (CNS) characterized by multiple lesions disseminated in time and space. Tumefactive MS is a rare variant of MS presenting with a large intracranial lesion, >2 cm in diameter with mass effect and perilesional edema and/or ring enhancement with gadolinium (1).

The rapidly progressive clinical deterioration of the MS patient presented here posed a diagnostic challenge requiring a multidisciplinary diagnostic workup. In the literature, various case reports describing the challenging diagnostic procedure of tumefactive MS due to varied clinical presentations as well as clinical courses can be found. Our report describes the diagnostic and therapeutic approach of a rare and at the same time extremely severe course of MS. It demonstrates that brain biopsy may be necessary for differential diagnosis in an immunocompromised MS patient with progressive brain lesions.

## Case Presentation

A 51-year-old man of Mediterranean origin with an 8-year history of relapsing-remitting MS (RRMS) was admitted to our hospital on suspicion of a relapse.

After diagnosis in 2009, the patient had initially been treated with glatiramer acetate. The family medical history offered that the patient's mother and uncle (blood related) both suffered from MS. The patient's uncle died at the age of 52 years after being bedridden for a longer time. The patient had four relapses under glatiramer acetate necessitating treatment with intravenous (IV) steroids initially with a good treatment response. The first relapse leading to the diagnosis of a clinically isolated syndrome (CIS) was an acute central vestibular syndrome leading to dizziness and an ataxic gait dysfunction. At this time, MRI already revealed multiple white matter lesions in the supratentorium, cerebellum, and cervical as well as thoracic spinal cord.

In 2012, owing to an increasing relapse rate and incomplete clinical remissions, the medication was changed to natalizumab. At this time, the last relapses under glatiramer acetate had led to a residual paraparesis with emphasis on the left and a left side internuclear ophthalmoplegia. At the last relapse, brain MRI scan of the brain showed multiple white matter lesions with a cystic aspect and incomplete ring-like gadolinium enhancement. After the medication was switched to natalizumab, the disease course stabilized, and he suffered no more relapses. However, when the anti-JC virus (JCV) antibody level index (Stratify™) rose to 4.5, natalizumab was discontinued early in 2017. Subsequently, fingolimod was started 3 months prior to admission and 4 weeks after discontinuation of natalizumab.

The first symptoms appeared 8 days before admission: a progressive left lower limb weakness and deterioration of walking ability became evident. At that time, the patient was able to stand without help and walk a few steps with unilateral assistance [Expanded Disability Status Scale (EDSS) 6.0].

**Figure 1:**
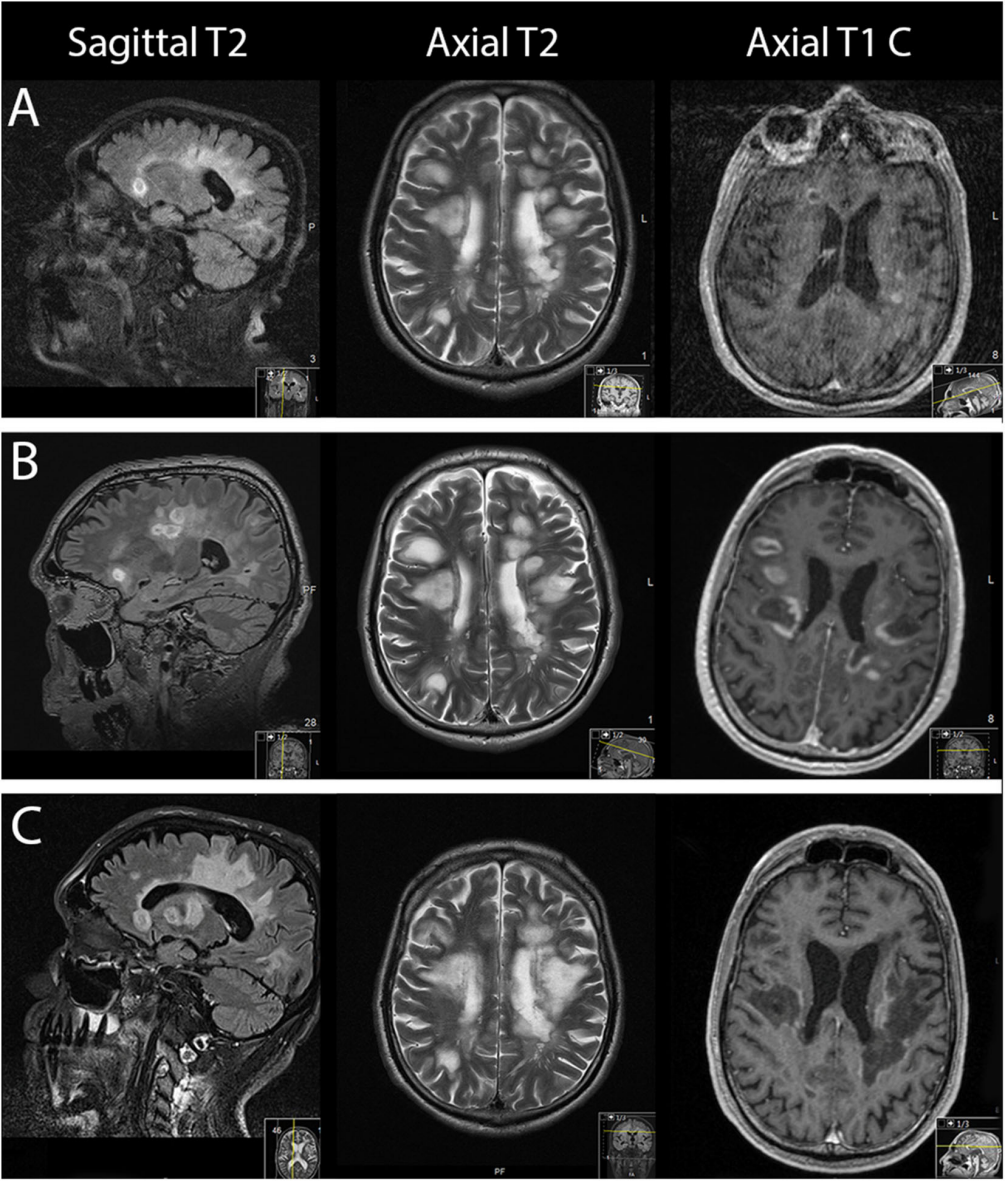
From left to right: sagittal, axial MRI T2-weighted sequences and axial gadolinium contrast T1-weighted sequences. **(A)** On the day of admission. **(B)** Three days after admission. **(C)** Forty-one days after admission.

At this time (after treatment with natalizumab and rising anti-JCV antibody level index), the differential diagnoses were an MS relapse or progressive multifocal leukoencephalopathy (PML). The MRI scan of the brain on the day of admission showed bihemispheric confluent T2 white matter lesions without changes, typical for PML (Figure 1A). Cerebrospinal fluid (CSF) analysis revealed a normal white blood cell count (2/μl) with mildly increased lactate and glucose levels. The albumin quotient was normal, but oligoclonal bands were positive with intrathecal synthesis of immunoglobulins G and M. Polymerase chain reaction (PCR), microbiological, and serological study findings were all negative (including JCV PCR, JCV CSF/serum antibody index, HSV-1 PCR, HSV-2 PCR, VZV PCR, EBV PCR, and HIV PCR). Evoked potentials revealed an impairment of the corticospinal tract to the right leg, bilaterally impaired tibial nerve somatosensory reactions, and evidence of a bilateral affection of the visual system.

On suspicion of an MS relapse, the patient was treated with IV methylprednisolone 1,000 mg once daily for five consecutive days. In response to this therapy, his walking ability slightly improved. However, 2 days later, the patient's clinical status dramatically worsened, necessitating his transfer to the intensive care unit (ICU): he became somnolent and mutistic, exhibiting a bilateral horizontal gaze palsy. In addition, he became tetraplegic and had bilaterally positive Babinski signs corresponding to an EDSS score of 9.5. MRI scan of the brain at that time showed progressive bihemispheric confluent white matter lesions (Figure 1B). The patient developed a severe aspiration pneumonia with respiratory failure requiring intubation and subsequent tracheostomy for mechanical ventilation.

The progressive white matter lesions were judged as the radiological correlate of a clinical MS relapse.

After antibiotic treatment for pneumonia had led to a reduction of the leukocytosis and C-reactive protein, IV steroids were applied for 6 days. However, as no clinical improvement was observed on IV steroids, seven cycles of plasma exchange (PLEX) were performed. Another follow-up MRI scan of the brain revealed further progression of gadolinium enhancement and T2 lesion load.

Despite high-dose IV steroids and PLEX, the clinical condition of the patient deteriorated further. With negative laboratory results, and radiological findings atypical for PML, other differential diagnoses had to be considered. Characteristic imaging findings in PML are one or more regions of FLAIR/T2 showing hyperintense confluent white matter lesions, inconsistent in size and shape, typically involving subcortical U-fibers and sparing the cortex, leading to a sharp border between lesion and cortex. MS lesions typically present a periventricular distribution, whereas PML lesions more commonly involve the subcortical white matter.

The following differential diagnoses were considered for our patient:

- tumefactive MS relapse,
- neurocysticercosis,
- neurosarcoidosis,
- intracerebral lymphoma,
- atypic PML, and
- other viral encephalitis.

The negative results of the CSF analysis and serology argued against a neurocysticercosis, lymphoma, or PML. Cysticercosis is the most common parasitic infection of the CNS. However, the presentation on MRI imaging was judged unusual. Electroencephalography (EEG) was normal. For sarcoidosis, a CT scan of the chest and laboratory tests for ACE and sIL2R were added; both proved negative. The negative CSF findings and radiological presentation also argued against an intracerebral lymphoma but did not definitely exclude one. A tumefactive MS relapse is a rare course and commonly presents with a large intracerebral lesion (>2 cm) with mass effect and perilesional edema and/or ring enhancement with gadolinium.

**Figure 2:**
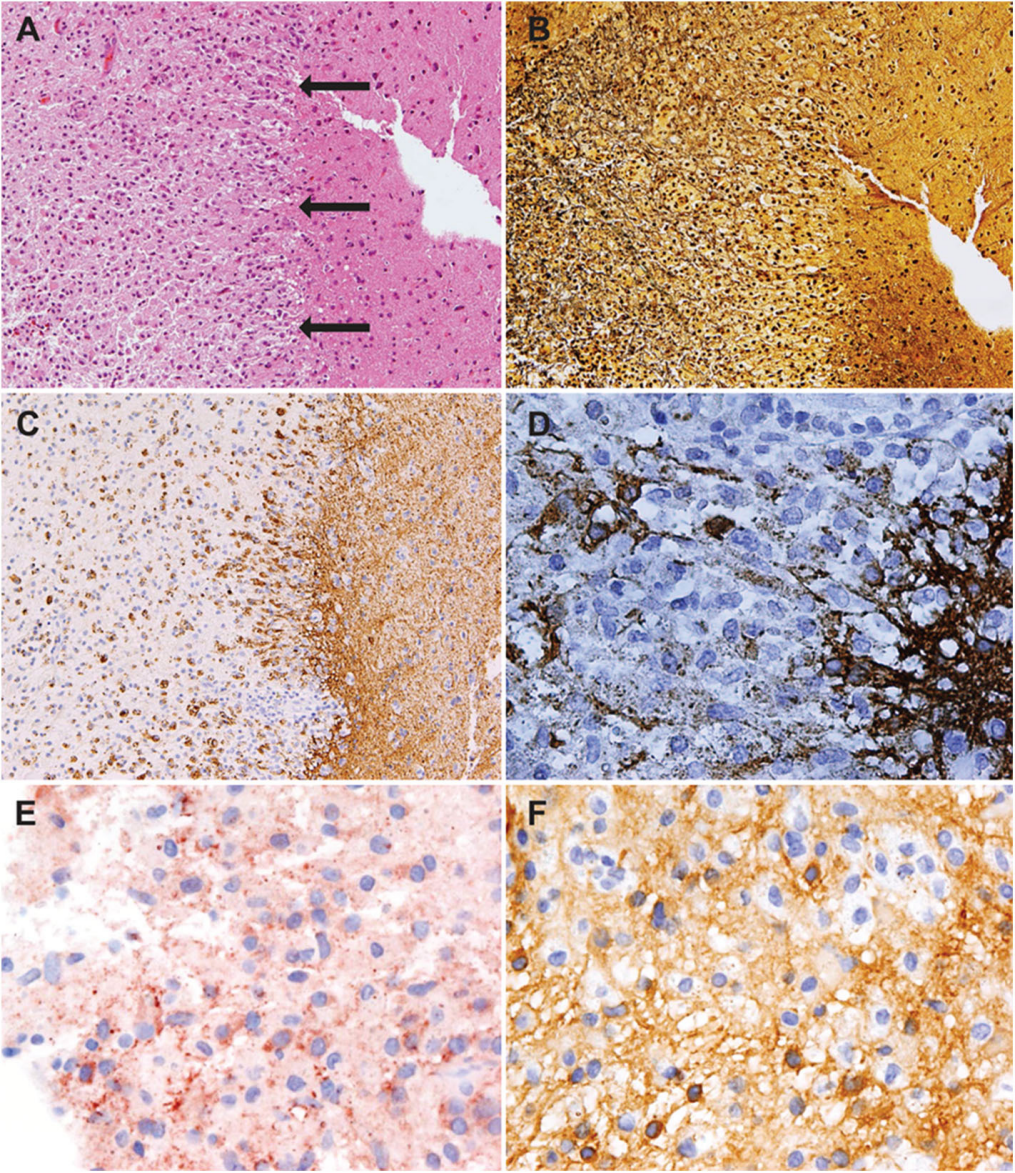
Histology showed an active demyelinating multiple sclerosis (MS) lesion corresponding to immunopathological pattern II. Arrows indicate the sharply demarcated inflammatory subcortical plaque on the left; the cerebral cortex is present on the right [H&E stain, ×10 **(A)**]. Axons were preserved within the lesion [Bielschowsky silver stain, ×10 **(B)**], whereas myelin was lost [proteolipid protein, ×10 **(C)**; cyclic nucleotide phosphodiesterase stain, ×40 **(D)**]. The lesion showed early active demyelination as indicated by the presence of major **(C)** and minor myelin proteins **(D)** within the macrophages. Complement [c9neo complement stain, ×40 **(E)**] and immunoglobulin G [IgG; IgG stain ×40 **(F)**] were found within the macrophages, suggesting a complement and immunoglobulin-mediated demyelination (pattern II).

To further differentiate between a tumefactive MS relapse and a less likely intracerebral lymphoma, a stereotactic biopsy of a lesion in the right frontal lobe was performed. The biopsy revealed a sharply demarcated inflammatory demyelinating lesion consistent with MS. Inflammatory infiltrates within the lesion consisted of CD3 dominated by CD8-positive T cells as well as CD138-positive plasma cells (Figure 2). Deposits of complement and immunoglobulins identified the lesion as an antibody/complement mediated type of MS, described previously as pattern II MS (2). PCR analysis revealed low copy numbers of human herpesvirus 6 (HHV-6) DNA in tissue (32 copies/μg DNA). However, axons were preserved, thus ruling out a necrosis. Also, no evidence was found for lymphoma.

Given the diagnostic uncertainty between HHV-6 encephalitis and tumefactive MS lesions, we opted for a polypragmatic approach and induced a therapy with IV foscarnet and ganciclovir (3) along with another course of high-dose IV steroids for 5 days. The final neuropathologic results were suggestive of a pattern II MS lesion according to Lassmann et al. (2).

The MRI scan of the brain after therapy showed progression of the T2 lesion load; however, the regression of gadolinium enhancement suggested remission of acute inflammation (Figure 1C). The neurological status remained severely impaired: the tetraplegia slightly improved by developing into a severe tetraparesis of 2/5 at the upper and persisting plegia of lower limbs (EDSS 8.5).

Subsequently, the patient was transferred to a neuro-rehabilitation center. His clinical status did not improve, even after 3 months of rehabilitation. Currently, the patient is tetraparetic and lives in a special-care home where he spontaneously breathes through tracheostomy and receives enteral long-term nutrition via percutaneous endoscopic gastrostomy (PEG).

After fingolimod was discontinued during the acute phase, the possibility of administering a new highly active preventive MS treatment such as ocrelizumab or alemtuzumab in order to avoid further relapses was discussed with the patient's relatives but was discarded owing to fear of infectious complications and the patient's poor clinical status and prognosis. Unfortunately, no further MRI imaging has been performed after discharge from our clinic.

## Discussion

The rapid clinical deterioration posed a diagnostic challenge. Because of increasing anti-JCV index upon treatment with natalizumab, we speculated that PML might have occurred. However, the MRI findings were atypical (uncommon mass effect and degree of gadolinium enhancement). CSF analyses including PCR, microbiological, and serological studies were all negative. In view of the absence of clinical improvement and radiological progression despite high-dose IV steroids and PLEX, further differential diagnoses were considered. However, CSF findings argued against neurocysticercosis, lymphoma, or PML. Negative CSF findings for JCV (PCR) did not completely exclude PML because viral loads can be very low (<100 copies/mL); the detection threshold of commercial tests is about 200 copies/mL. With no signs of mediastinal lymphadenopathy in the CT scan of the chest and nonelevated serum ACE and sIL2-R levels, neurosarcoidosis seemed less likely. The negative CSF findings as well as the radiological presentation made the possibility of an intracerebral lymphoma or HHV-6 encephalitis less likely but could not rule them out. Therefore, a stereotactic biopsy was needed. The histopathological results yielded an inflammatory demyelinating MS lesion with low titers of HHV-6 DNA quantified by PCR. There were no morphological features of a necrotizing HHV-6-encephalitis. In correlation with low copies of HHV-6-DNA, an HHV-6 encephalitis seemed very unlikely.

HHV-6 belongs to the *Herpesviridae* family. In the general population, virus latency in adenoid tissues/tonsils is almost 100%, usually acquired during childhood. Cells persist in a latent viral state mainly in leukocytes and can directly integrate their DNA into host cells (4). The prevalence of integrated HHV-6 DNA in healthy blood donors is 0.5% (5). Thus, detection in a blood sample does not definitely indicate an active infection. The same applies to infection and replication in the CNS; studies have revealed the presence of HHV-6 DNA even in healthy brain tissues (6).

HHV-6 encephalitis is characterized by necrotizing brain lesions and typically high copy numbers of virus DNA in CSF and/or brain tissue. Although the disease is a rare event, it should be taken into account in an immunocompromised patient with necrotizing brain lesions. Brain biopsy should be considered because it is important that patients receive a proper diagnosis in order to possibly benefit from an antiviral therapy combining foscarnet and ganciclovir (3). In this context, advanced imaging modalities like FET-PET or MRI spectroscopy may be of help and should be further investigated for their usefulness in making the differential diagnosis.

## Conclusions

In our patient, the results of the brain biopsy finally confirmed the diagnosis of a tumefactive MS lesion. All pathological features of MS were fulfilled including inflammatory demyelination, relative axonal preservation, and gliosis. The early active demyelinating nature of the lesion allowed us to classify the lesion as having the immunopathological pattern II, which has recently been shown to improve clinically in 55% in response to apheresis therapy (7). Our case exemplifies that treatment response can be dramatically negative. Possible explanations include that the onset of PLEX at 13 days after relapse onset may have been too late and/or that the damage was too severe.

Severe MS rebounds after highly effective treatment with fingolimod as well as natalizumab were previously reported (8). The differentiation between a recurrence of disease activity and a rebound, implying a more severe disease course than before natalizumab treatment, is difficult. On histological grounds, the present biopsy showed an MS lesion with an inflammatory infiltrate not exceeding the MS typical inflammation. However, a rebound can be assumed based on the clinical and MRI findings. Switching from natalizumab to fingolimod might increase the risk of tumefactive MS (9). Predictive factors to identify risk groups are warranted.

## Data Availability Statement

The original contributions presented in the study are included in the article/supplementary material, further inquiries can be directed to the corresponding author/s.

## Ethics Statement

Written informed consent was obtained from the individual(s) for the publication of any potentially identifiable images or data included in this article.

## Author Contributions

KB gave the idea of case reporting, analyzed the case, and drafted the manuscript for intellectual content. AA revised the figures and critically reviewed the manuscript. MF, NG, HH, OA, and H-PH critically reviewed the manuscript. BK prepared the MRI scans as figures and critically reviewed the manuscript. DH critically reviewed the manuscript and revised the MRI sequences for interpretation. BT critically reviewed the manuscript and interpreted the MRI sequences. IM, WB, and GR critically reviewed the manuscript and interpreted the neuropathology results. PA critically reviewed the manuscript. All authors contributed to the article and approved the submitted version.

## Conflict of Interest

The authors declare that the research was conducted in the absence of any commercial or financial relationships that could be construed as a potential conflict of interest.

## Abbreviations

ACE: angiotensin-converting enzyme
CNS: central nervous system
CSF: cerebrospinal fluid
EBV: Epstein–Barr virus
EDSS: expanded disability status scale
EEG: electroencephalography
FET-PET: ^18^F-fluoro-ethyl-tyrosine positron emission tomography
HHV-6: human herpesvirus 6
HIV: human immunodeficiency virus
HSV-1/HSV-2: herpes simplex virus type 1/type 2
ICU: intensive care unit
IV: intravenous
JCV: John Cunningham virus
MRI: magnet resonance imaging
MS: multiple sclerosis
PCR: polymerase chain reaction
PEG: percutaneous endoscopic gastrostomy
PLEX: plasma exchange
PML: progressive multifocal leukoencephalopathy
RRMS: relapsing-remitting multiple sclerosis
sIL-2-R: soluble interleukin-2-receptor
VZV: varicella zoster virus.
